# Hemi-Hemimegalencephaly or Posterior Quadrantic Dysplasia, a Rare Cause of Focal Epilepsy in an Otherwise Healthy Young Woman: A Case Report

**DOI:** 10.7759/cureus.10002

**Published:** 2020-08-24

**Authors:** Alexandre Feidert

**Affiliations:** 1 Department of Radiology, CUB Hôpital Erasme/Université Libre de Bruxelles (ULB), Brussels, BEL

**Keywords:** hemimegalencephaly, focal hemimegalencephaly, malformations of cortical developement, focal seizures, partial epilepsy, mri, epilepsy, seizures, neuroradiology

## Abstract

Hemimegalencephaly (HME) and its more localized form - posterior quadrantic dysplasia (PQD) - are rare malformations of cortical development (MCD) that normally manifest as refractory focal epilepsy and cognitive impairment in children. We report a case study of a 19-year-old woman who presented with seizure-like symptoms to the emergency department after discontinuing her seizure treatment having fled her country. MRI revealed typical signs of PQD. This case study demonstrates how an unusual mild clinical presentation led to the late diagnosis of this rare MCD.

## Introduction

Hemimegalencephaly (HME) is a rare congenital malformation of cortical development (MCD) characterized by enlarged and dysplastic hamartomatous overgrowth of one or all parts of a cerebral hemisphere [1,2]. HME may be present in isolation or conjunction with other neurocutaneous syndromes such as epidermal nevus, Klippel-Trénauany-Weber syndrome, neurofibromatosis type 1, hypomelanosis of Ito, Proteus syndrome, or more rarely, tuberous sclerosis [3,4]. The MRIs of patients with HME reveal moderate-to-marked enlargement of an entire or a part of a cerebral hemisphere with the normal, dysplastic, or heterotopic cortex. Moreover, the cortical-white matter junction may appear indistinct with variable degrees of T1 and T2 signal intensities due to heterotopia and astrocytosis. The ipsilateral ventricles appear enlarged with a characteristic shape of a frontal horn that seems to point anteriorly and superiorly [1]. Recent research studies have identified pathway gene mutations in the PIK3-AKT-MTOR pathway as a potential etiopathogenesis for MCD and other malformations, and it could possibly be a target for future treatment options [5,6]. Clinical manifestations of HME and other MCDs vary depending on the severity of the malformation, and they commonly present as medical refractory epilepsy and cognitive impairment in the pediatric population and are rarely reported in the adult population [3,7]. Other clinical manifestations could be early feeding problems, infantile spasm, macrocephalus, hydrocephalus, and mild hemiparesis [2]. Most commonly, HME affects an entire hemisphere, but localized forms have also been reported in the literature, with the most notable being posterior quadrantic dysplasia (PQD) or hemi-HME with an enlargement of the parieto-occipito-temporal lobe, sparing the frontal lobe of a single hemisphere [4,8-11]. Dysmorphic ipsilateral occipital horn with high-signal posterior periventricular white matter and abnormal gyri can be observed in most patients with PQD [2,8].

In this study, we report the case of a 19-year old woman suffering from hemi-HME who was referred to our institution for neurosurgical counseling after visiting an emergency department after experiencing symptoms of fainting.

## Case presentation

**Figure 1:**
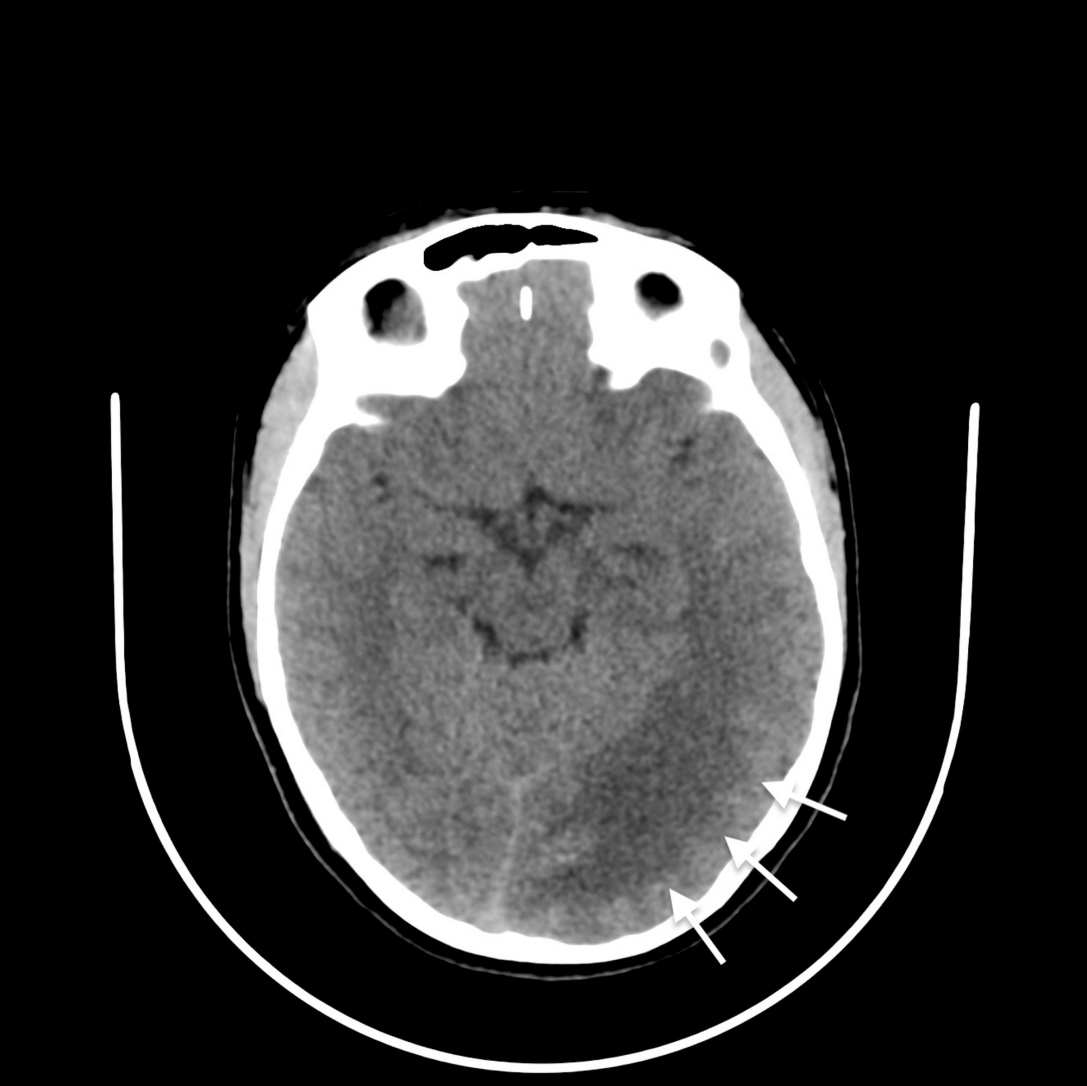
Non-contrast enhanced computed tomography of the head Enlarged hypodense left parieto-occipital lobe with a “mass-like” effect (white arrows).

A 19-year old woman visited the emergency department for the treatment of pre-syncopal symptoms without loss of consciousness and with flu-like symptoms. However, due to the presence of a linguistic barrier, the first language of the patient being Syrian and the physician speaking French, the collection of initial patient history was limited. Moreover, bloodwork did not indicate any significant anomalies; in particular, there were no signs of infection or inflammation. Furthermore, clinical examination was unremarkable and no signs of neurological deficits could be established. Non-enhanced CT, which was performed to rule out a central cause, revealed the presence of a large hypodense “mass-like” lesion in the left parieto-occipital region (Figure 1). Finally, the patient was transferred to the neurosurgical department of our institution for further clinical and imaging workup.

**Figure 2:**
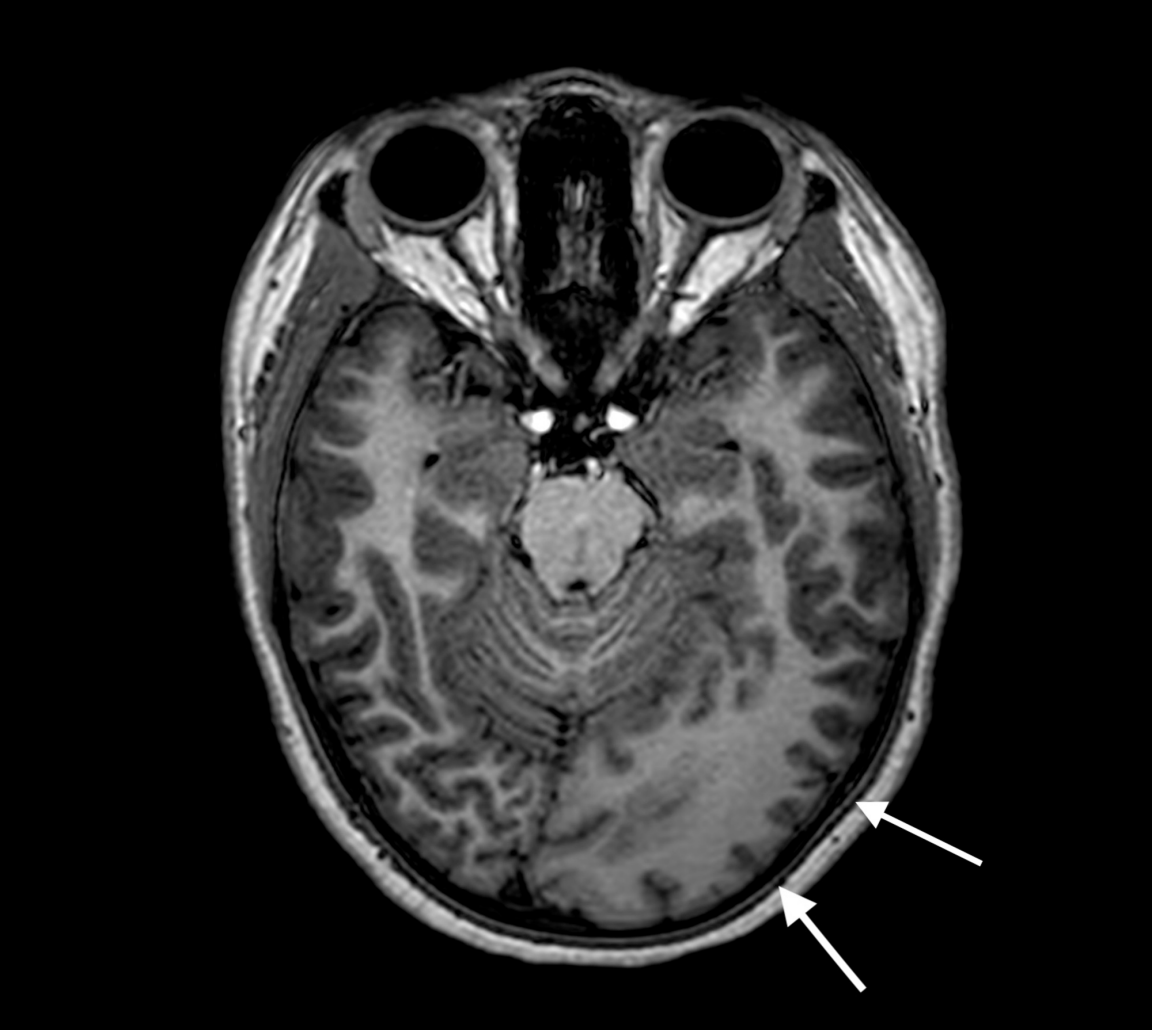
Three-dimensional T1 weighted sequence with axial reformatting Enlarged left occipital lobe with a cortex with decreased signal intensity (white arrows).

**Figure 3:**
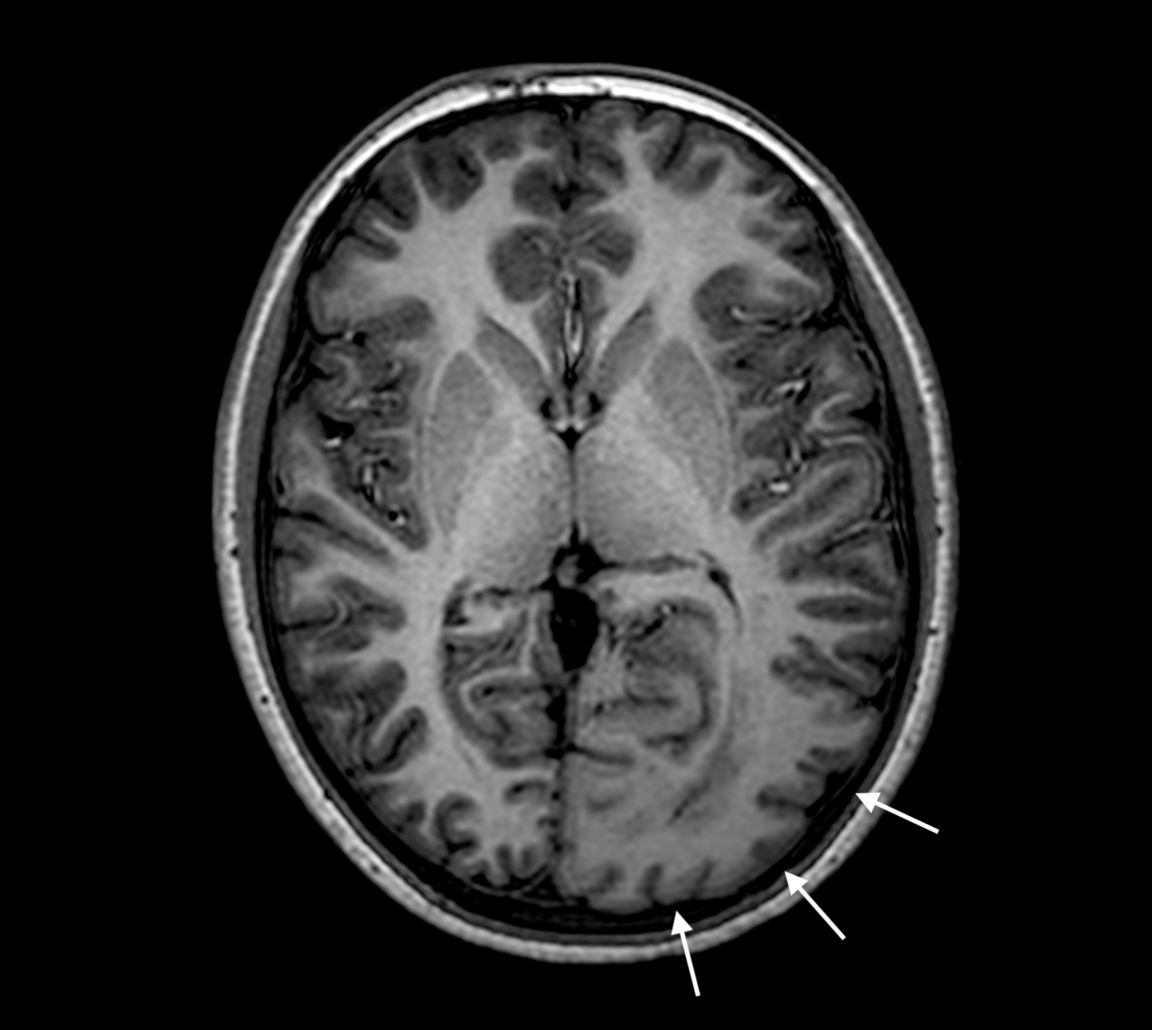
Three-dimensional T1 weighted sequence with axial reformatting Enlarged left parieto-occipital lobe with a cortex with decreased signal intensity (white arrows).

**Figure 4:**
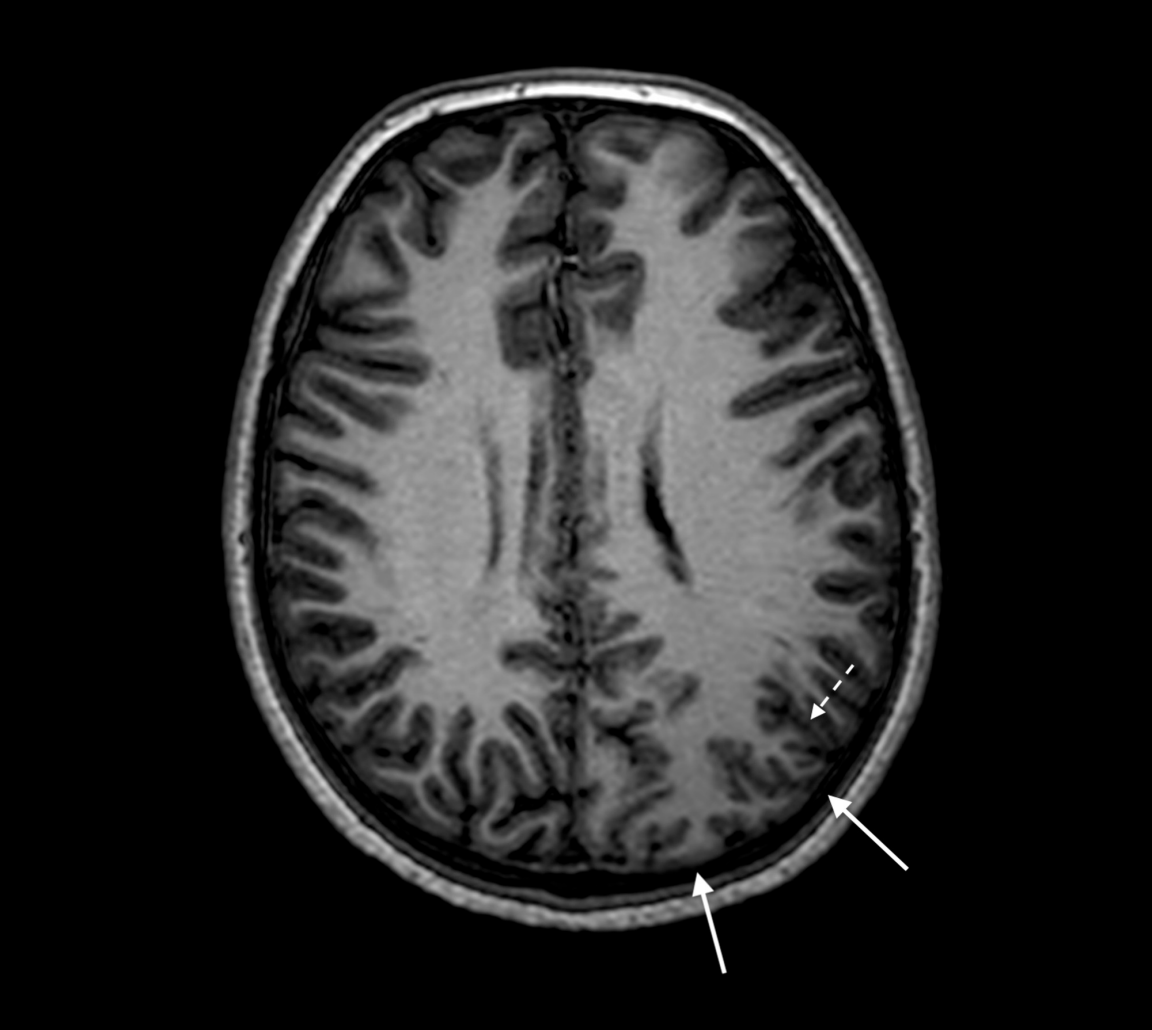
Three-dimensional T1 weighted sequence with axial reformatting Enlarged left parietal lobe (white arrows) with polymicrogyria pattern and cortex with decreased signal intensity (dotted white arrow).

**Figure 5:**
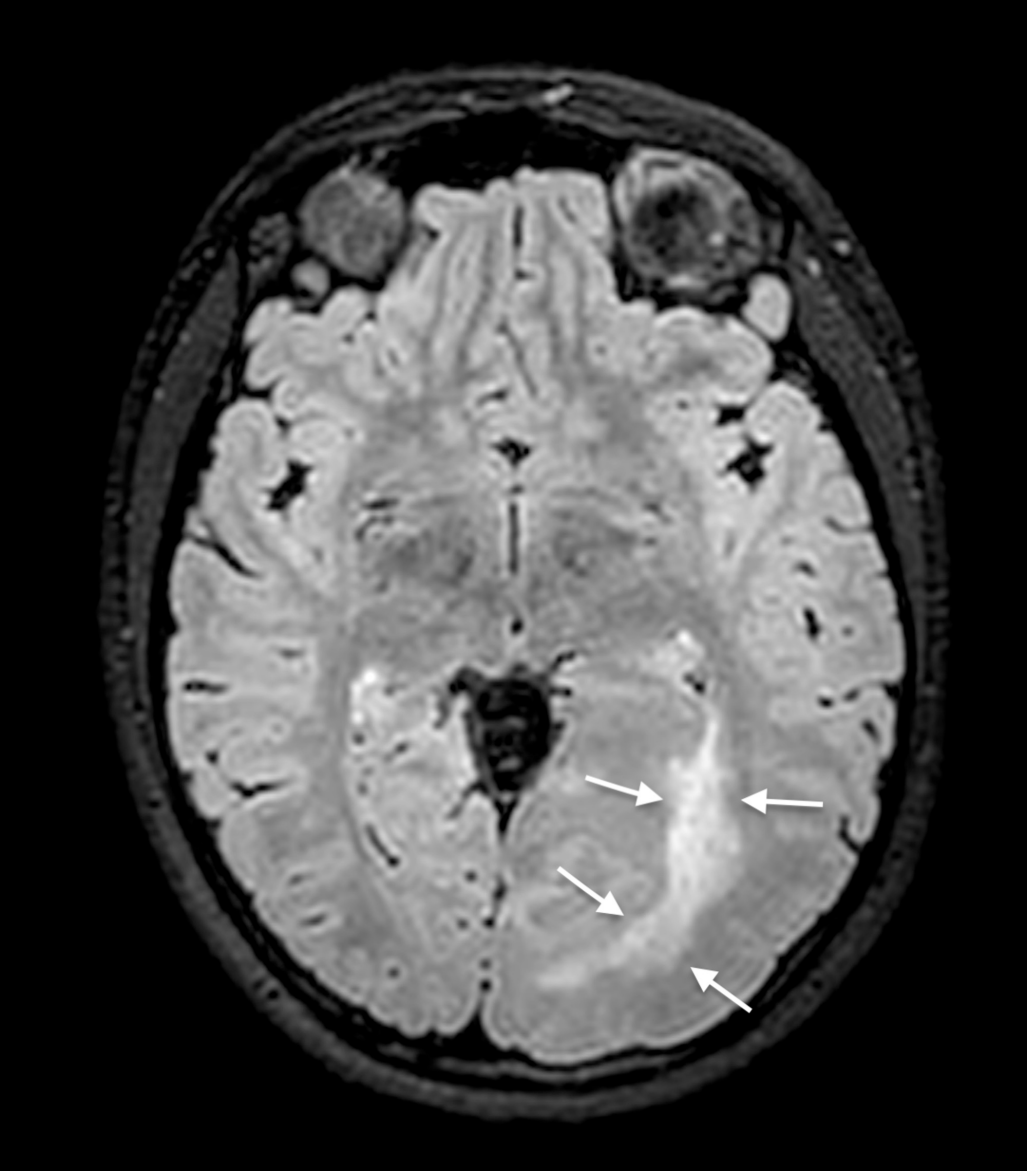
Three-dimensional FLAIR sequence with axial reformatting Increased signal intensities around the left occipital horn consistent with astrogliosis (white arrows). FlAIR: fluid-attenuated inversion recovery.

**Figure 6:**
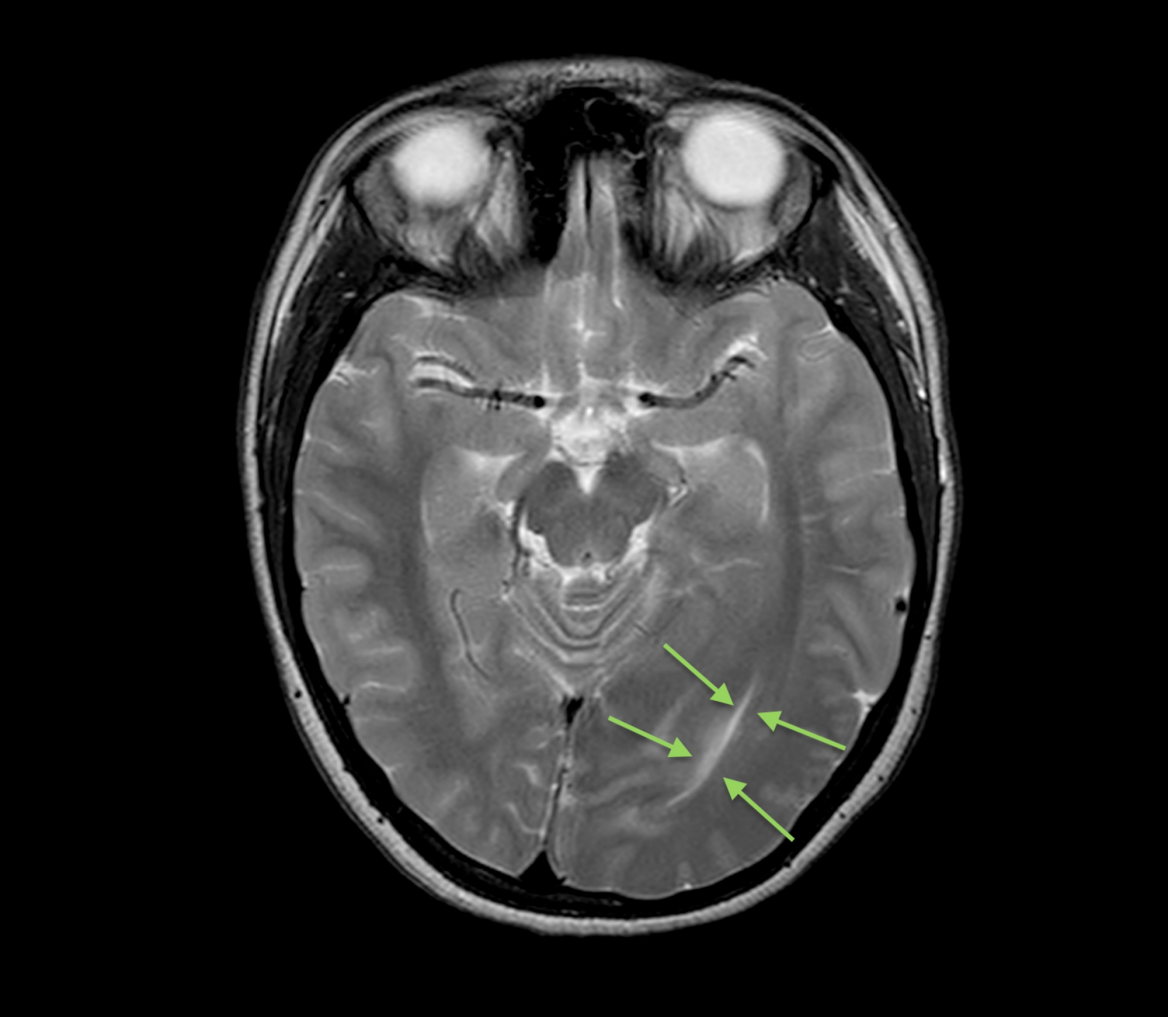
Axial T2 weighted sequence Enlarged left occipital horn (green arrows).

**Figure 7:**
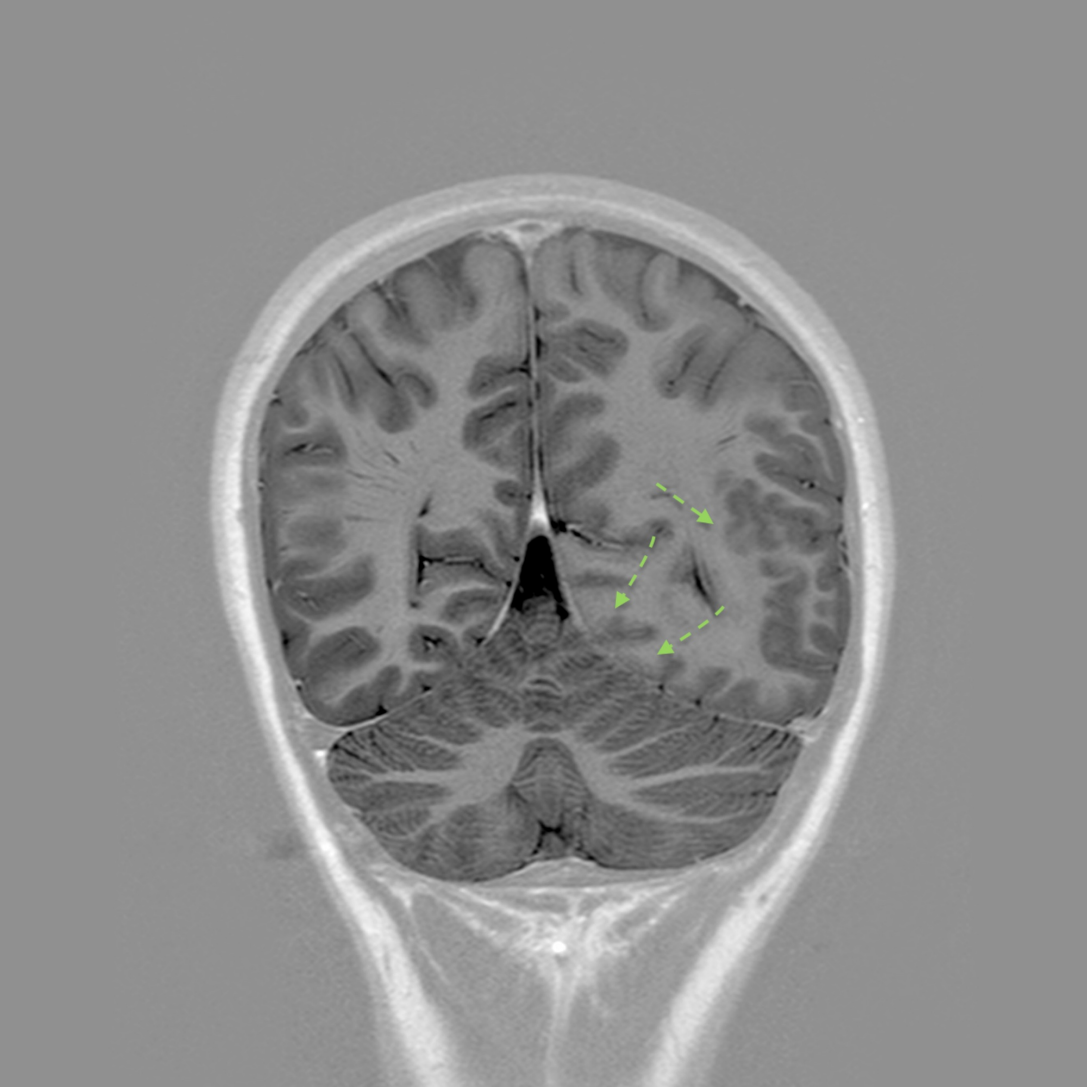
Coronal T1 inversion-recuperation sequence Diffuse-reduced signal intensity cortex with blurred cortical-white matter junctions in the left parieto-occipital lobe (dotted green arrows).

**Figure 8:**
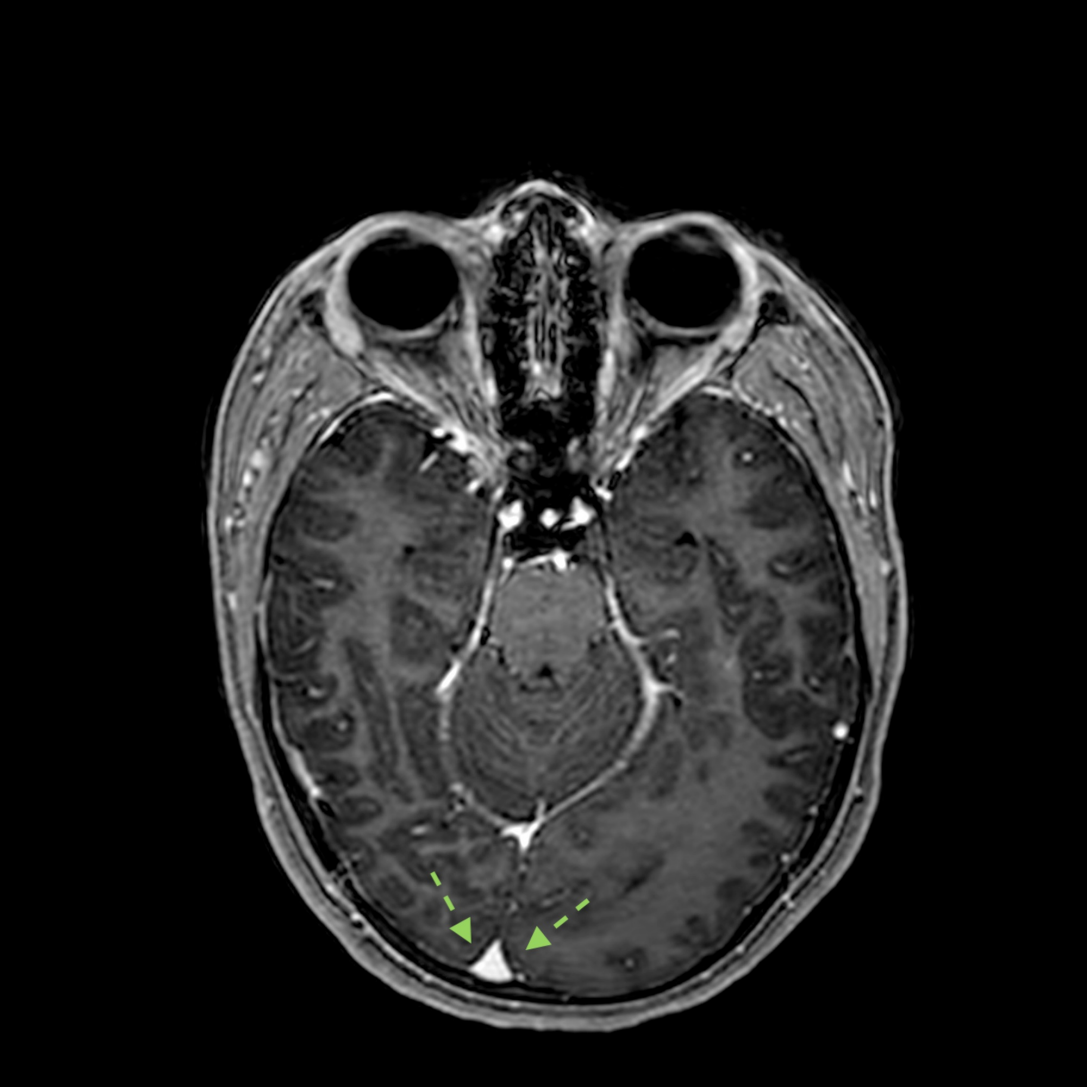
Three-dimensional T1 after I.V. contrast sequence with axial reformatting No contrast uptake in the parieto-occipital lobe and right deviation of the sagittal longitudinal sinus (dotted green arrows). I.V.: intravenous.

MRI performed on a 3T Philips Achieva MRI (Koninklijke Philips N.V., Amsterdam, the Netherlands) showed marked enlargements of the left parieto-occipital lobe and left occipital horn, periventricular astrogliosis, and polymicrogyria with blurred cortical-white matter junction that is consistent with the diagnosis of PQD (Figures 2-8). 

A translator assisted with the heteroanamnesis and revealed that the patient was born at home and had apparently experienced hypoxia during birth. Her mother had told her that she was blue for two days after birth; however, no further workup or hospitalization was done. Nonetheless, thereafter, her psychomotor development appeared to be normal. However, she then suffered her first seizure at the age of 8. The seizures appeared as bilateral tinnitus, which was followed by loss of consciousness and spasms that lasted for five to seven minutes. For this condition, she was medicated with lamotrigine, which reduced the frequency of seizures from a few times a week to once a year. However, her treatment had to be discontinued, as she had to flee her country because of war at the age of 16.

Electroencephalography revealed a highly active epileptogenic focus with a spike-and-wave pattern in the left occipital region, which was suggestive of cortical dysplasia.

Subsequently, treatment was reinitiated with a gradual increase in the dose of lamotrigine to 75 mg twice a day. Moreover, no notable adverse effects were reported. The patient still remains seizure-free after one year of treatment.

## Discussion

Our case study presents classical MRI findings that are consistent with the diagnosis of PQD or hemi-HME. However, the clinical presentations were mild compared to the extent of the brain malformation. Moreover, seizures were easily controlled by a medical treatment, which led to the late discovery of PQD.

Typically, patients with HME and PQD experience early-onset intractable seizures, which are poorly controlled by medical therapy, often requiring surgical interventions such as hemispherectomy [7,12].

HME is a rare condition, accounting for only 3% of cortical dysplasia diagnosed by imaging [13]. However, the precise prevalence of hemi-HME remains uncertain, with most papers in the literature consisting of case reports, the largest of which included 14 patients [8].

Best diagnostic clues are obtained with MRI, due to its superior performance in imaging gray and white matter and its non-irradiating nature.

## Conclusions

Although HME and PQD are rare MCD, characteristic findings on MRI should be known to the radiologist. HME and PQD commonly affect the pediatric population and initial diagnosis in adults are much more infrequently reported.
